# Frustrated Alternative Approaches towards the Synthesis of a Thermally Stable 1,2-Diazacyclobutene

**DOI:** 10.3390/molecules29174068

**Published:** 2024-08-28

**Authors:** Gary W. Breton, Kenneth L. Martin

**Affiliations:** Department of Chemistry and Biochemistry, Berry College, 2277 Martha Berry HWY, Mount Berry, GA 30149, USA; kmartin@berry.edu

**Keywords:** 1,2-diazete, 1,2-dihydrodiazete, 1,2-diazacyclobutene, 1,2-diazetidine, 1,2-diazacyclobutane

## Abstract

We have previously demonstrated that an appropriately substituted four-membered-ring 1,2-diazacyclobutene is a useful compound in organic synthesis for the introduction of strained 1,2-diazetidine rings. In order to further explore the reactivity of this interesting heterocycle, we sought a method to improve upon the poor synthetic yield reported earlier. A novel route involving the synthesis of a similarly substituted 1,2-diazetidine compound followed by free-radical bromination and base-catalyzed debromination appeared promising. While there are some studies on the synthesis of the desired 1,2-diazetidine precursor, when we attempted its synthesis, we instead observed the exclusive formation of an eight-membered “dimer”-like compound. The structure of this compound was confirmed via single-crystal X-ray analysis. Fortunately, an alternative synthetic approach for the formation of the desired 1,2-diazetidine precursor proved successful, and the structure of the precursor has been confirmed via X-ray analysis. However, unfortunately, the required bromination step proved to be more challenging than expected, and ultimately, this route had to be abandoned since the anticipated improvement upon the original yield did not seem promising. Single-crystal X-ray analysis proved pivotal in properly identifying the structures of the synthesized compounds.

## 1. Introduction

**Scheme 1:**
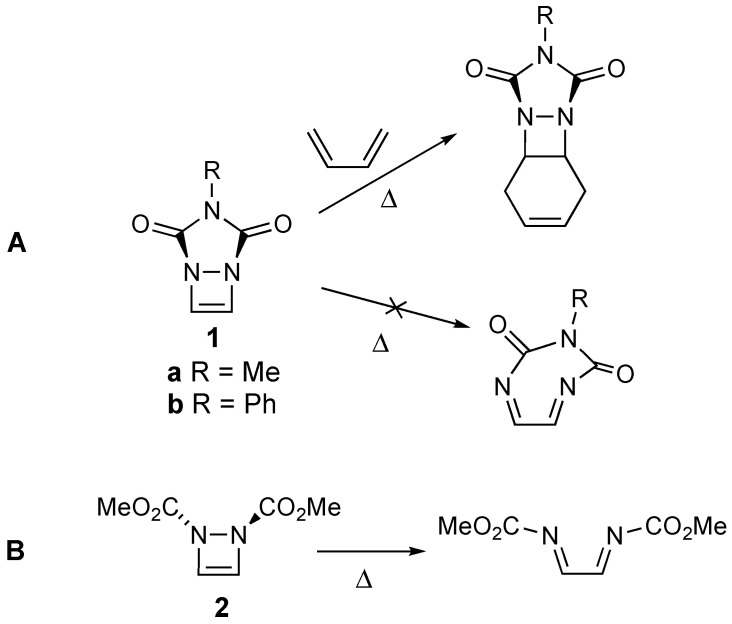
(**A**) Reactivity of diazetine **1** via Diels–Alder reactions to form 1,2-diazetidines, and its resistance to electrocyclic ring opening; (**B**) Thermally allowed electrocyclic ring opening of diazetine **2**.

Several years ago, we introduced 1,2-diazacyclobutene **1a** (Scheme 1) as a convenient dienophile for the integration of the strained 1,2-diazetidine motif into molecular structures via Diels–Alder reactions (see Scheme 1A) [1,2,3].

The fused urazole ring that is part of the structure of **1a** proved critical to the thermal stability of the 1,2-diazacyclobutene ring system. In the absence of this fused ring, a thermally allowed conrotatory electrocyclic ring opening occurs, even at ambient temperatures, as has been reported for compound **2** (Scheme 1) [4]. The fused urazole ring prevents the ring opening process due to the strain that would be imposed on the resulting ring-opened product (see Scheme 1A) [1]. While **1a** successfully engaged in Diels–Alder reactions with a variety of dienes, the utility of the synthetic method was limited by the low overall yield (15%) for the three-step synthesis of **1a** [1].

**Scheme 2:**
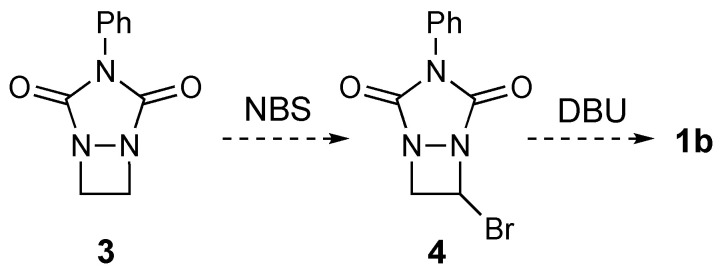
Proposed synthesis of **1b**.

To further investigate the reactions of 1,2-diazacyclobutenes such as **1** in a more expansive manner, we realized that the development of a more robust, higher-yielding synthetic method was necessary. Therefore, we considered the possibility of synthesizing the *N*-Ph derivative **1b**, as shown in Scheme 2. This novel synthetic route required the synthesis of the saturated 1,2-diazetidine compound **3**. Earlier work found in the literature suggested that the synthesis of **3** was plausible [5,6,7]. Upon the successful synthesis of **3**, we envisioned that routine free-radical bromination of **3** by NBS would afford 3-bromo-1,2-diazetidine **4**. Finally, the base-catalyzed elimination of HBr from **4** using standard E2 conditions (e.g., DBU in DMF) would afford the desired product **1b**. Assuming the synthesis of **3** (see below) and that of the two subsequent steps proceeded in reasonable yields, we hoped this new route might be of higher overall yield than the previous route followed for **1a**. In this paper, we describe our attempts at the synthesis of compounds **3** and **4**, which proved to be more challenging than anticipated, and illustrate the importance of X-ray crystallography for the confirmation of structures.

## 2. Results and Discussion

**Scheme 3:**
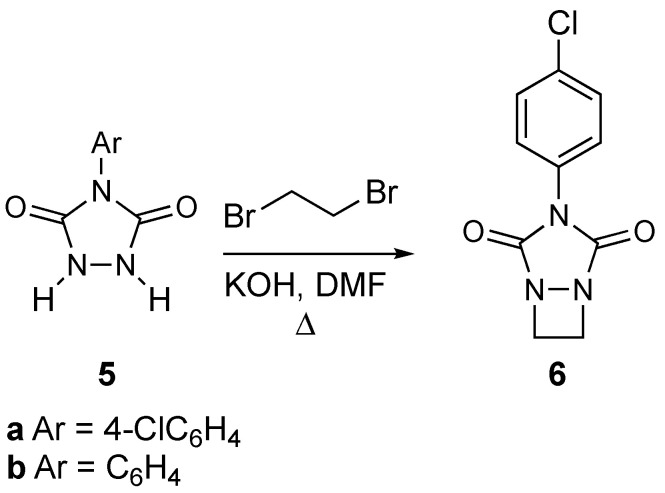
Literature procedure [5] for the formation of **6** (Ar = 4-ClC_6_H_6_) as extended towards the synthesis of **3**.

We began by attempting to synthesize 1,2-diazetidine **3**. The synthesis of a derivative of **3** had already been reported (see **6** in Scheme 3) via the double nucleophilic substitution of the potassium salt formed from urazole **5a** with 1,2-dibromoethane [5]. Although the reported yield of **6** was low (21% yield), the availability and low cost of the starting materials (we opted to use commercially available urazole **5b** rather than **5a**) and the operational simplicity for the synthesis of **3** made it an attractive approach. Following the previous literature, a solution of 4-phenyl urazole (**5b**), KOH, and 1,2-dibromoethane was heated in DMF to afford a small amount (9% yield) of a white solid product. We were heartened that the ^1^H and ^13^C NMR data appeared to be consistent with the structure of the anticipated product, **3**, including a singlet for all four methylene protons in the ^1^H NMR spectrum (δ 4.14), and a total of six carbons in the ^13^C NMR spectrum, reflecting the symmetry of the ring system. However, we thought that the compound’s melting point of 252–253 °C was unexpectedly high for such a small molecule. On the other hand, compound **6** was also reported to have a high melting point (>280 °C) [5].

**Figure 1:**
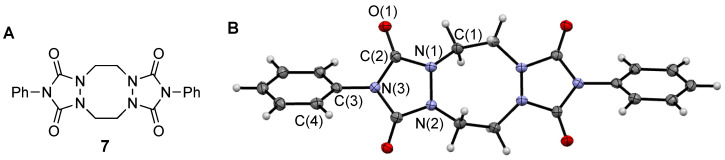
(**A**) The structure of **7** and (**B**) an ORTEP representation of the X-ray crystal structure of **7** with thermal ellipsoids drawn at 50% probability. Selected bond lengths (Å): N(1)-N(2), 1.420; N(1)-C(1), 1.480; N(1)-C(2), 1.383, C(2)-N(3), 1.388; N(3)-C(3), 1.434; and C(2)-O(1), 1.208. Selected bond angles (°): C(1)-N(1)-N(2), 118.1; C(1)-N(1)-C(2), 118.2; and N(2)-N(1)-C(2), 108.0. Selected dihedral angles (°): N(2)-N(1)-C(2)-N(3), 10.8 and C(2)-N(3)-C(3)-C(4), 138.7.

Therefore, for conclusive verification of the structure, a crystal suitable for X-ray analysis was grown. Surprisingly, the X-ray analysis revealed the structure to be not that of the desired compound **3** but that of the “dimerized” eight-membered ring compound **7** instead (see Figure 1).

**Scheme 4:**
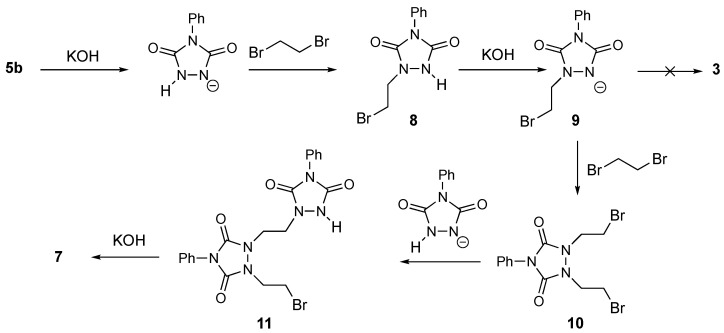
Proposed mechanism for the formation of dimer compound **7**.

The formation of compound **7** can be rationalized as shown in Scheme 4. In the presence of the base KOH, *N*-phenylurazole **5b** is deprotonated due to its low pKa value of approximately 5 [8]. Nucleophilic attack onto 1,2-dibromoethane affords the monoalkylated compound **8**. Deprotonation of **8** affords anion **9**. Anion **9** can either undergo intramolecular substitution to afford diazetidine **3**, or it could react with a second molecule of dibromide to form **10**. Apparently, a bimolecular reaction to form the relatively unstrained compound **10** is favored over the intramolecular cyclization that would form the strained structure of **3**. This is also in line with the known general reluctance of compounds to undergo intramolecular reactions to afford four-membered rings [9]. Once formed, **10** can undergo further reaction to afford **11** followed by an intramolecular cyclization that yields the observed compound **7**.

Attempts to circumvent the dimerization process via changes to the reaction conditions, such as running the reaction under more dilute conditions (in an attempt to discourage the formation of **10**) or changing the base to Cs_2_(CO_3_)_2_ instead of KOH, failed to produce any product other than **7**. Note that the structure for **7** is much more in line with the previously mentioned high melting point and further suggests that the structure for compound **6** may be misassigned in the literature.

**Scheme 5:**
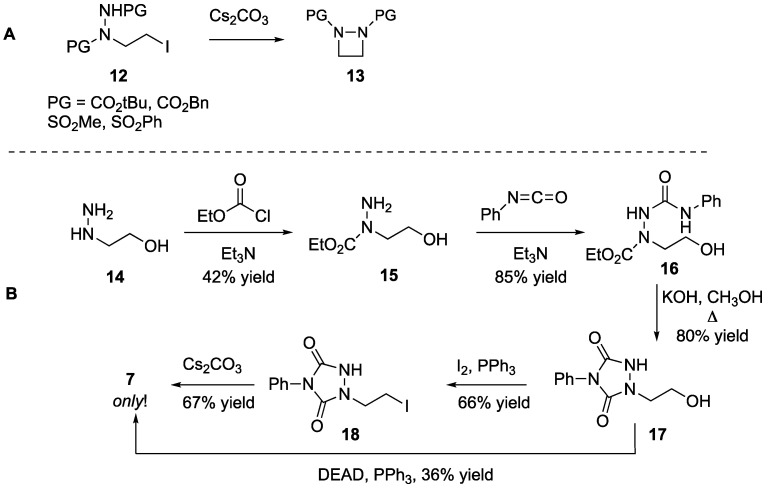
(**A**) Shipman’s [6] cyclization of protected hydrazines (**12**) to form diazetidines (**13**). (**B**) Attempted extension of Shipman’s synthetic method to form **3**.

Given the failure of the above method to secure the desired compound **3**, we took an alternative approach. Shipman had reported the syntheses of *N*,*N-*diprotected 1,2-diazetidines such as **13** via a cyclization reaction starting from halogenated precursor **12** (Scheme 5A) [6]. We considered the possibility of extending this methodology toward the synthesis of compound **3**, as shown in Scheme 5B. Thus, the treatment of commercially available 2-hydroxyethylhydrazine **14** with ethyl chloroformate provided **15**. A reaction between **15** with phenyl isocyanate gave **16**, which could be easily cyclized to form 2-hydroxyethyl-*N*-phenylurazole **17**. We attempted the final ring closure of **17** by two methods. First, iodination of alcohol **17** provided **18**. Unfortunately, once more, the treatment of **18** according to the procedure worked out earlier by Shipman [6] for the synthesis of **13** did not yield the desired compound **3** and only yielded the “dimerized” **7**. Similarly, an attempted direct Mitsunobu-type [10] intramolecular reaction of alcohol precursor **17** also only afforded **7**.

**Scheme 6:**
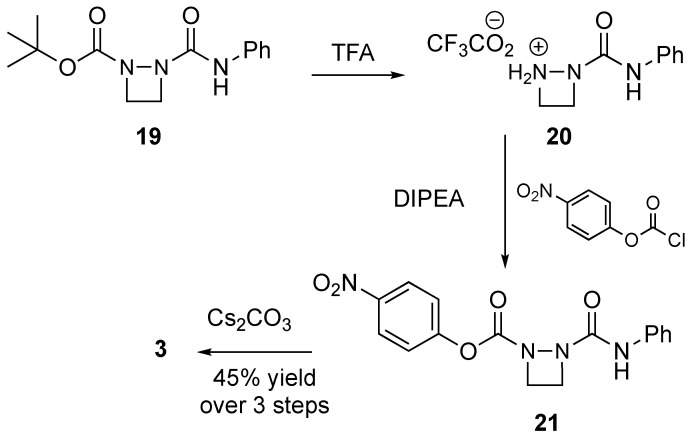
Successful synthesis of **3** starting from previously reported compound **19** [7].

It was interesting that these cyclizations failed even though compound **13** could be formed from precursor compound **12**. We surmised that the reason for the failure of the ring closure of **17** and **18** may be due to the rigidity of the urazole ring structure. The rigidity of the ring structure might inhibit the intramolecular nucleophilic attack that is necessary to form **3**. Therefore, we worked out a new alternative synthetic scheme that would install the urazole ring structure *after* the cyclization process (see Scheme 6). Hence, starting with an already intact diazetidine compound **19** that had been previously reported by Shipman [7], we removed the BOC group with TFA. The treatment of the resulting crude trifluoroacetate salt **20** with 4-nitrophenyl chloroformate in the presence of the base diisopropylethylamine afforded **21**. Crude **21** was then successfully cyclized with Cs_2_(CO_3_)_2_ to afford the desired diazetidine **3** as a white solid in 45% yield over three steps. The melting point of this compound, 173–174 °C, was much more in line with what would be expected.

**Figure 2:**
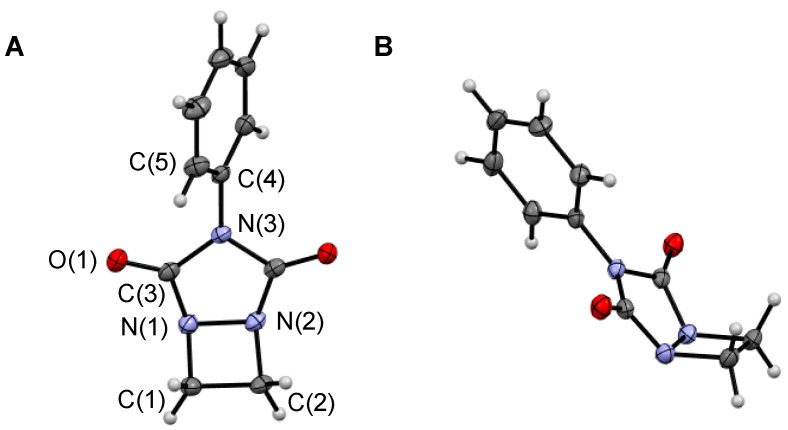
ORTEP representation of the X-ray crystal structure of compound **3** with thermal ellipsoids drawn at 50% probability as visualized (**A**) from the front and (**B**) from the side of the structure. Selected bond lengths (Å): C(1)-C(2), 1.538; C(1)-N(1), 1.510; N(1)-N(2), 1.481; N(1)-C(3), 1.395; C(3)-O(1), 1.210; C(3)-N(3), 1.396; and N(3)-C(4), 1.435. Selected bond angles (°): C(1)-N(2)-C(3), 121.0; C(1)-N(1)-N(2), 91.5; and N(1)-C(3)-N(3), 105.9. Select dihedral angles (°): C(1)-C(2)-N(2)-N(1), 1.9 and C(3)-N(3)-C(4)-C(5), 53.2.

The ^1^H and ^13^C NMR spectra of both **3** and **7** are provided in the Supplementary Materials. Despite the significant difference in the structures of these two compounds, the spectra are very similar. Not surprisingly, the aromatic regions are nearly identical in both cases, and there is only a subtle shift of the signals for the methylene protons for **3** and **7** in the ^1^H NMR spectra (singlets at 4.47 and 4.14 ppm, respectively) and in the ^13^C NMR spectra (51.6 and 47.8 ppm, respectively). Therefore, wary of being deceived again, as in the case of compound **7**, we verified the structure of **3** via X-ray analysis. This time, however, the data were in complete agreement with the structure of the desired compound **3** (Figure 2).

**Figure 3:**
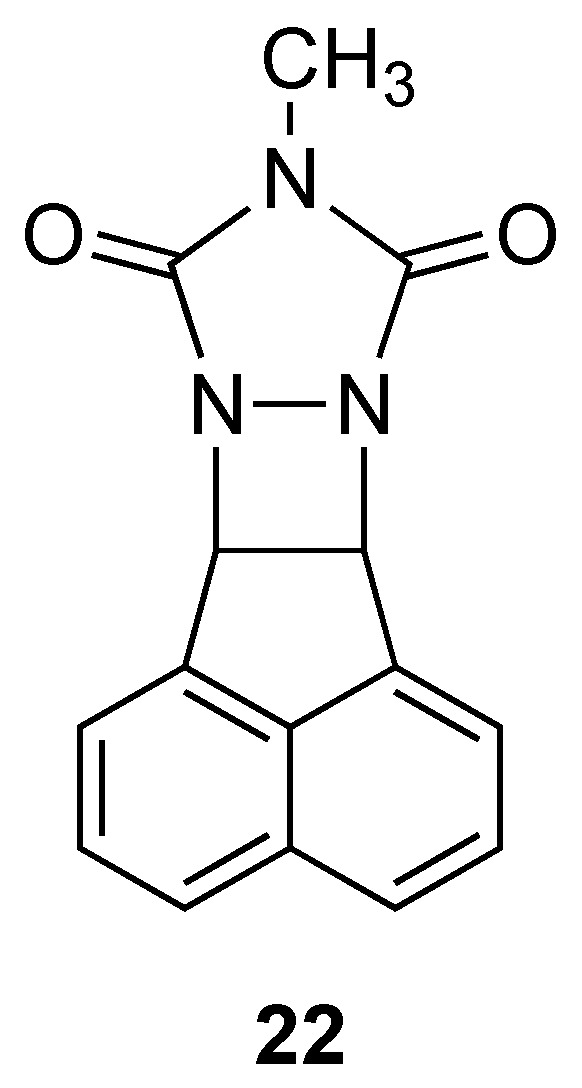
Structure of previously reported diazetidine **22**.

The *N*-*N* bond in the urazole ring of **3** (1.481 Å) was observed to be longer than the corresponding bond in dimer **7** (1.420 Å). It was also longer than the corresponding bond for **13** (PG = CO_2_tBu) of 1.450 Å [6], as well as previously reported **1a** (1.470 Å) [11]. However, it was nearly identical to the bond length (1.480 Å) for the *N*-*N* bond of the similarly substituted diazetidine **22** (Figure 3) [12]. Interestingly, the bend of the urazole ring in relation to the four-membered ring, as measured by the C-N-(CO) bond angle, was 121.0° for **3**, which was remarkably similar to that of both diazacyclobutene **1a** (119.8°) and diazetidine **22** (122.6°), suggesting a preferred degree of nitrogen atom pyramidalization bound within a four-membered ring regardless of the type of carbon to which it is attached (i.e., SP^2^ in **2** versus SP^3^ in **3** and **22**, respectively).

Having finally obtained **3**, we were free to pursue its free-radical bromination in pursuit of compound **4** (see Scheme 2). However, unfortunately, the bromination of this substrate proved difficult. Using standard bromination conditions (NBS, benzoyl peroxide, and heat [13]) upwards of 30% (estimated based on the ^1^H NMR spectrum) of the desired compound **4** could be formed based on the observation of chemical shifts consistent with the anticipated structure. In particular, the singlet representing the four methylene protons for compound **3** was transformed into three individual multiplets at 6.29 (C*H*-Br), 5.12, and 4.61 ppm, which was consistent with monobromination. In addition, conducting a ^13^C NMR experiment revealed two different saturated carbons (64.0 and 61.4 ppm) instead of the single carbon observed for compound **3** (51.6 ppm). The signal at 64.0 ppm was determined to be a CH carbon and the signal at 61.4 ppm was determined as a CH_2_ carbon by a DEPT experiment. However, unfortunately, attempts at further bromination did not appear to increase the yield and instead led to the formation of complex product mixtures. This was surprising since we expected that the nitrogen atom within the urazole ring would stabilize the radical intermediate formed during the bromination process and, thereby, promote the reaction [14]. However, in this case, it appears to have the opposite effect. Therefore, given the number of steps required to form **3**, in addition to the reluctance of **3** to cleanly brominate to form **4**, we felt it prudent to abandon this route towards the synthesis of diazetine **1b**. However, other synthetic pathways are currently being pursued in our labs.

## 3. Materials and Methods

### 3.1. General Methods

Column chromatography was performed on a silica gel absorbent (234–400 mesh). Thin-layer chromatography was performed on silica gel plates that were pre-coated with a fluorescent indicator. Developed TLC plates were visualized by ultraviolet light. ^1^H and ^13^C NMR spectra were obtained on a JEOL NMR spectrometer at 400 and 200 MHz, respectively. All chemical shifts are reported in units of parts per million downfield from TMS. Reported high-resolution mass spectra (HRMS) data were acquired via the sampling technique of electron spray ionization utilizing an LTQ-FTMS hybrid mass spectrometer. Unless otherwise stated in the Experimental Procedures section, all compounds were purchased from commercial sources and used as received.

### 3.2. Experimental Procedures

#### 3.2.1. Synthesis of “Dimer” **7** from Urazole **5b**

Reaction following the literature procedure [5]: to a solution of 2 g (0.11 mole) of *N*-phenylurazole (**5b**) in 30 mL of anhydrous DMF, 0.74 g (1 eq) of powdered KOH was added. The reaction mixture was stirred for 2 h until all the KOH went into solution. 1,2-Dibromoethane (2.07 g, 1 eq) was then added to the solution via pipette, and the reaction mixture was heated to 100 °C for 0.5 h. It was then further heated to reflux for 1 h. After cooling to room temperature, the reaction mixture was filtered, and the collected salts were rinsed with 10 mL of DMF. The DMF was removed using a rotary evaporator to yield a thick liquid. Water (50 mL) was added to the thick liquid, and a precipitate formed. The mixture was extracted with 2 × 30 mL of CH_2_Cl_2_. The combined organic layers were backwashed with 2 × 30 mL of H_2_O, dried over Na_2_SO_4_, filtered, and concentrated to afford a thick pale orange-brown liquid. Column chromatography (SiO_2_, 10% methanol in CH_2_Cl_2_) afforded 183 mg (8% yield) of **7** as a white solid, m.p. 251–252 °C: ^1^H NMR (400 MHz, CDCl_3_) δ 7.49–7.51 (m, 8H), 7.42 (h, 2H), 4.14 (s, 8H); ^13^C{^1^H} NMR (100 MHz, CDCl_3_)153.9, 131.1, 129.5, 128.8, 125.5, 47.9; HRMS (ESI) *m*/*z* [M+H]+ Calcd for C_20_H_19_N_6_O_4_ 407.14623; Found 407.14514.

Reaction under dilute conditions: to a solution of 2 g (0.11 mole) of *N*-phenylurazole (**5b**) in 60 mL of anhydrous DMF, 0.74 g (1 eq) of powdered KOH was added. The reaction mixture was stirred for 2 h until all the KOH went into solution. 1,2-Dibromoethane (2.07 g, 1 eq) was then added to the solution via a pipette, and the reaction mixture was heated to 100 °C for 0.5 h. It was then further heated to reflux for 1 h. After cooling to room temperature, the reaction mixture was filtered, and the collected salts were rinsed with 10 mL of DMF. The DMF was removed using a rotary evaporator to yield a thick liquid. The analysis of this reaction mixture by ^1^H NMR spectroscopy revealed only the presence of compound **7**.

Reaction using Cs_2_CO_3_ as a base: to a solution of 0.5 g (2.82 mmole) of *N*-phenylurazole (**5b**) in 10 mL of anhydrous DMF, 1.1 g (1.2 eq) of powdered Cs_2_CO_3_ was added. The reaction mixture was stirred for 1 h, and then, 1,2-dibromoethane (0.53 g, 1 eq) was added to the solution via a pipette, and the reaction mixture was heated to 100 °C for 0.5 h. The reaction mixture was then further heated to reflux for 1 h. After cooling to room temperature, the reaction mixture was filtered, and the collected salts were rinsed with 10 mL of DMF. The DMF was removed using a rotary evaporator to yield a thick liquid. The analysis of this reaction mixture by ^1^H NMR spectroscopy revealed only the presence of compound **7**.

#### 3.2.2. Synthesis of **11**

To 2 g (0.026 mol) of 2-hydroxyethylhydrazine in 15 mL of CH_2_Cl_2_, 3.26 mL (1 eq) of Et_3_N was added via a syringe. The resulting solution was cooled to 0 °C, and 2.82 g (1 eq) of ethyl chloroformate was added dropwise over the course of 10 min. A thick white precipitate formed. The resulting mixture was stirred for an additional 10 min and then warmed to room temperature. After 4 h of stirring at room temperature, the mixture was poured into 50 mL of THF and then filtered to remove the precipitate. The solvent was removed via rotary evaporation to provide a thick oil. Column chromatography (SiO_2_, 5% methanol in EtOAc) afforded 1.62 g (42% yield) of **11** as a clear oil: ^1^H NMR (400 MHz, CDCl_3_) δ 4.17 (q, 2H), 3.8–4.4 (br s, 3H), 3.82 (t, *J* = 4.8 Hz, 2H), 3.60 (t, *J* = 4.8 Hz, 2H), 1.28 (t, 3H); ^13^C{^1^H} NMR (100 MHz, CDCl_3_) 158.0, 62.1, 60.8, 51.9, 14.6; HRMS (ESI) *m*/*z* [M+H]+ Calcd for C_5_H_13_N_2_O_3_ 149.09207; Found 149.09183.

#### 3.2.3. Synthesis of **12**

To 1.53 g of **11** (10.3 mmol) in 15 mL of benzene, 1.23 g (1 eq) of phenyl isocyanate was added dropwise. The solution became cloudy. The mixture was then heated to reflux for 5 h, cooled to room temperature, and concentrated to a thick liquid. Column chromatography (SiO_2_, 5% methanol in EtOAc) afforded 2.34 g (85% yield) of **12** as a clear oil. The NMR spectra reflected a mixture of 2 slowly interconverting conformers, which severely complicated the spectra. Signals for the major isomer are provided: ^1^H NMR (400 MHz, DMSO-D_6_) δ 8.14 (br s, 1H), 7.60 (br s, 1H), 7.36 (br d, *J* = 7.5 Hz, 2H), 7.21 (br t, *J* = 7.5 Hz, 2H), 7.00 (br t, *J* = 8.5 Hz, 1H), 4.12 (br q, *J* = 7.2 Hz, 2H), 3.74 (br s, 4 H), 1.21 (br t, *J* = 7.2 Hz, 3H); ^13^C{^1^H} NMR (100 MHz, CDCl_3_) δ 157.6, 157.5, 138.1, 129.0, 123.6, 119.8, 63.1, 58.8, 53.3, 14.5; HRMS (ESI) *m*/*z* [M+H]+ Calcd for C_12_H_18_N_3_O_4_ 268.12918; Found 268.12850.

#### 3.2.4. Synthesis of **13**

To 2.34 g (8.75 mmol) of **12** in 10 mL of CH_3_OH, 1.44 g (2.5 eq) of KOH pellets was added. The stirring mixture was heated to reflux for 3 h. After cooling to room temperature, the solution was diluted with 10 mL of H_2_O and acidified to pH~2 with concentrated HCl (~3 mL). The resulting clear colorless solution was concentrated into a free-flowing white solid. This solid was extracted with 1 × 75 mL and 2 × 50 mL of boiling EtOAc. Concentration of the combined EtOAc solutions afforded 1.54 g (80% yield) of **13** as a pale yellow crystalline solid, m.p. 161–162 °C: ^1^H NMR (400 MHz, DMSO-D_6_) δ 10.8 (br s, NH, 1H), 7.42–7.57 (m, 4H), 7.39 (t, *J* = 7.0 Hz, 1H), 4.90 (br s, OH, 1H), 3.63 (t, *J* = 5.0 Hz, 2H), 3.3.57 (t, *J* = 5.0 Hz, 2H); ^13^C{^1^H} NMR (100 MHz, DMSO-D_6_) δ 152.8, 152.4, 132.2, 129.0, 127.9, 126.2, 57.5, 48.4; HRMS (ESI) *m*/*z* [M+H]+ Calcd for C_10_H_12_N_3_O_3_ 222.08732; Found 222.08660.

#### 3.2.5. Synthesis of **14**

To a solution of 0.66 g (2.53 mmol) of PPh_3_ and 0.17 g (2.53 mmol) of imidazole in 10 mL of CH_2_Cl_2_ cooled to 0 °C, 0.64 g (2.53 mmol) of solid I_2_ was added in portions over several minutes with stirring. The I_2_ eventually went into solution, resulting in the formation of a yellow-orange precipitate. This mixture was warmed to room temperature, and 0.47 g (2.11 mmol) of solid **13** was added in portions. The resulting mixture was stirred overnight and filtered. The separated solid was rinsed with CH_2_Cl_2_, and the filtrate was concentrated into a dark viscous liquid. Column chromatography (SiO_2_, 100% EtOAc) afforded 0.46 g (66% yield) of **14** as a crystalline white solid, m.p. 115–116 °C: ^1^H NMR (400 MHz, DMSO-D_6_) δ 10.9 (br s, NH, 1H), 7.43–7.53 (m, 4H), 7.37–7.43 (m, 1H), 3.87 (t, *J* = 6.4 Hz, 2H), 3.45 (t, *J* = 6.4 Hz, 2H); ^13^C{^1^H} NMR (100 MHz, DMSO-D_6_) δ 152.3, 152.2, 131.8, 128.9, 127.9, 126.1, 47.8, 2.0; HRMS (ESI) *m*/*z* [M+H]+ Calcd for C_10_H_11_N_3_O_2_^127^I 331.98905; Found 331.98819.

#### 3.2.6. Attempted Cyclization of **14**

To urazole **14** in 15 mL of dry DMF, solid Cs_2_CO_3_ was added, and the reaction mixture was stirred for 24 h. The residual solid was filtered under vacuum, and the filtrate was poured into a mixture of 25 mL H_2_O and 25 mL CH_2_Cl_2_ in a separatory funnel. The layers were mixed, and the organic layer was removed. The aqueous layer was washed with an additional 2 × 20 mL CH_2_Cl_2_, and the combined organic layers were backwashed with 1 × 25 mL H_2_O and 1 × 25 mL sat. aq. NaCl. The organic layer was dried and concentrated to afford 0.172 g of a white solid. The NMR spectra and m.p. of this compound matched that for “dimer” **7**, as described above.

#### 3.2.7. Attempted Cyclization of **13**

To a solution of 0.22 g (1 mmol) of **13** and 0.66 g (2.5 eq) of PPh_3_ in 100 mL of anhydrous THF, 1.14 mL of a 40 wt.% solution of DEAD in toluene (2.5 eq) was added. The resulting solution was stirred for 24 h and then concentrated. Column chromatography (SiO_2_, 10% methanol in CH_2_Cl_2_) afforded 76 mg (36% yield) of a white solid identified by NMR and m.p. as “dimer” **7**.

#### 3.2.8. Synthesis of **3**

To a solution of 0.5 g (1.81 mmol) of **15** in 10 mL of CH_2_Cl_2_, 1.25 mL of TFA was added using a syringe. The solution was stirred for 24 h and then concentrated to a sticky pale brown solid (crude compound **16**). To this solid in 5 mL of CH_2_Cl_2_, 0.63 mL (2 eq) of diisopropylethyl amine was added, followed by the dropwise addition of 0.36 g of para-nitrophenyl chloroformate. After stirring overnight, the reaction mixture was washed with 2 × 20 mL 0.5 N aq. HCl, dried over Na_2_SO_4_, filtered, and concentrated to 0.64 g of a pale brown solid (crude compound **17**). To this crude product in 50 mL of CH_3_CN, 1.22 g (approximately 2 eq) of Cs_2_CO_3_ was added. The solution became deep yellow almost immediately. After stirring for 3 hr, the reaction mixture was poured into 50 mL of CH_2_Cl_2_ and washed with 2 × 25 mL of 0.5 N aq. NaOH. The organic layer was dried over Na_2_SO_4_, filtered, and concentrated to a brown solid. Column chromatography (SiO_2_, 5% methanol in CH_2_Cl_2_) afforded 0.17 g (45% yield over the three steps) of **3** as a crystalline white solid, m.p. 173–174 °C: ^1^H NMR (400 MHz, CDCl_3_) δ 7.47–7.55 (m, 4H), 7.38–7.44 (m, 1 H), 4.47 (s, 4 H); ^13^C{^1^H} NMR (100 MHz, CDCl_3_) δ 163.0, 131.5, 129.4, 128.8, 125.3, 51.6; HRMS (ESI) *m*/*z* [M+H]+ Calcd for C_10_H_10_N_3_O_2_ 204.07675; Found 204.07615.

#### 3.2.9. Attempted Bromination of **3**

To 20 mg (0.09 mmol) of **3** in 0.5 mL of CDCl_3_, 44 mg (2.5 eq) of NBS (one equivalent was insufficient, and more than 2.5 equivalents led to complex reaction mixtures) and a few tiny crystals of benzoyl peroxide were added. The reaction mixture was irradiated by two nearby 300 W incandescent bulbs, the heat from which was sufficient to bring the reaction to a boil. After irradiating for 6 h, irradiation was stopped, and the reaction mixture was allowed to cool. The analysis of the reaction mixture by ^1^H NMR spectroscopy revealed new signals consistent with the monobromination of the ring, i.e., δ 6.29 (dd, *J* = 6.2, 4.2 Hz, 1 H), 5.12 (dd, *J* = 11.2, 6.2 Hz, 1 H), 4.61 (dd, *J* = 11.2, 4.2 Hz, 1 H). Integration relative to the starting material signal indicated approximately 26% conversion. However, further irradiation only increased the yield to approximately 37% while the starting material remained present and other competing products began to form.

### 3.3. X-ray Data Collection

X-ray diffraction data for **3** were measured at 100 K on a Rigaku XtaLAB AFC11 (RCD3) diffractometer with a rotating anode CuKα source (λ = 1.54184 Å) and a hybrid pixel array detector. X-ray diffraction data for **7** were measured at 100 K on a Rigaku XtaLAB Synergy-S diffractometer with a PhotonJet CuKα source (λ = 1.54184 Å) and HyPix-6000HE detector. Both structures were solved using *olex2.solve* [15] and refined using *olex2.refine* [16]. Hydrogen atoms were found in difference maps, and all parameters (position and isotropic temperature factor) were allowed to be refined. The absolute structure for **3** was found with a Flack parameter of −0.03(5). The absolute structure for **7** (a racemic twin with a BASF parameter of 0.38(18)) was found with a Hooft parameter of 0.04(11). The crystal and refinement data are presented in Table 1.

## Figures and Tables

**Table 1:** Crystal data and refinement parameters.

	3	7
Chemical formula	C_10_H_9_N_3_O_2_	C_20_H_18_N_6_O_4_
*M*_r_	203.20	406.40
Deposition number	2373853	2373840
Crystal system and space group	orthorhombic, *P*2_1_2_1_2_1_	monoclinic, *P*2_1_
Temperature (K)	100	100
*a*, *b*, *c* (Å)	6.00967(7), 11.74365(12), 13.52523(14)	7.8071(3), 7.1290(3), 16.3695(6)
β (°)	90	92.298(3)
*V* (Å^3^)	954.549(17)	910.34(6)
*Z*	4	2
Radiation type	Cu Kα	Cu Kα
μ (mm^−1^)	0.85	0.89
Crystal size (mm)	0.14 × 0.10 × 0.09	0.36 × 0.23 × 0.20
Diffractometer	XtaLAB AFC11 (RCD3)	XtaLAB Synergy-S
Absorption correction	Numerical and empirical	Numerical and empirical
*T*_min_, *T*_max_	1.000, 0.823	1.000, 0.585
No. of measured, independent, and observed [*I* > 2σ(*I*)] reflections	13,937, 1858, 1858	15,905, 3762, 3762
*R*_int_	0.0234	0.0533
(sin θ/λ)_max_ (Å^−1^)	0.615	0.633
*R*[*F*^2^ > 2σ(*F*^2^)], *wR*(*F*^2^), *S*	0.0233, 0.0553, 1.166	0.0344, 0.0854, 1.095
No. of reflections	1858	3762
No. of parameters	172	345
Δρ_max_, Δρ_min_ (e Å^−3^)	0.12, −0.19	0.20, −0.21

## Data Availability

Data supporting the reported results can be found in the Supplementary Materials. Additionally, CCDC 2355187 and 2355188 contain the supplementary crystallographic data for this paper. These data can be obtained free of charge via www.ccdc.cam.ac.uk/data_request/cif or by emailing data_request@ccdc.cam.ac.uk or by contacting the Cambridge Crystallographic Data Centre, 12 Union Road, Cambridge CB2 1EZ, UK; fax: +44 1223 336033.

## Supplementary Materials

The following supporting information can be downloaded at: https://www.mdpi.com/article/10.3390/molecules29174068/s1, CIF files for compounds **3** and **7** and a PDF file containing ^1^H and ^13^C NMR spectra for all new compounds.
