# Supplementary material for: Frustrated Alternative Approaches towards the Synthesis of a Thermally Stable 1,2-Diazacyclobutene
Source: Molecules. 2024 Aug 28;29(17):4068. doi: 10.3390/molecules29174068 (PMC11397526; doi:10.3390/molecules29174068)

Frustrated Alternative Approaches Towards the Synthesis of a Thermally Stable 1,2-Diazacyclobutene

Gary W. Breton * and Kenneth L. Martin

Supplementary Materials

^{1}H and ^{13}C NMR Spectra of Newly Characterized Compounds……………………..S2–S7

^{1}H NMR Spectrum of Compound 7 in $CDCl_3$ (400 MHz)

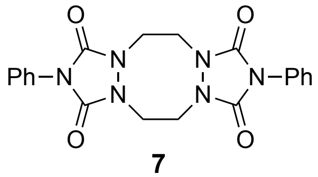

7

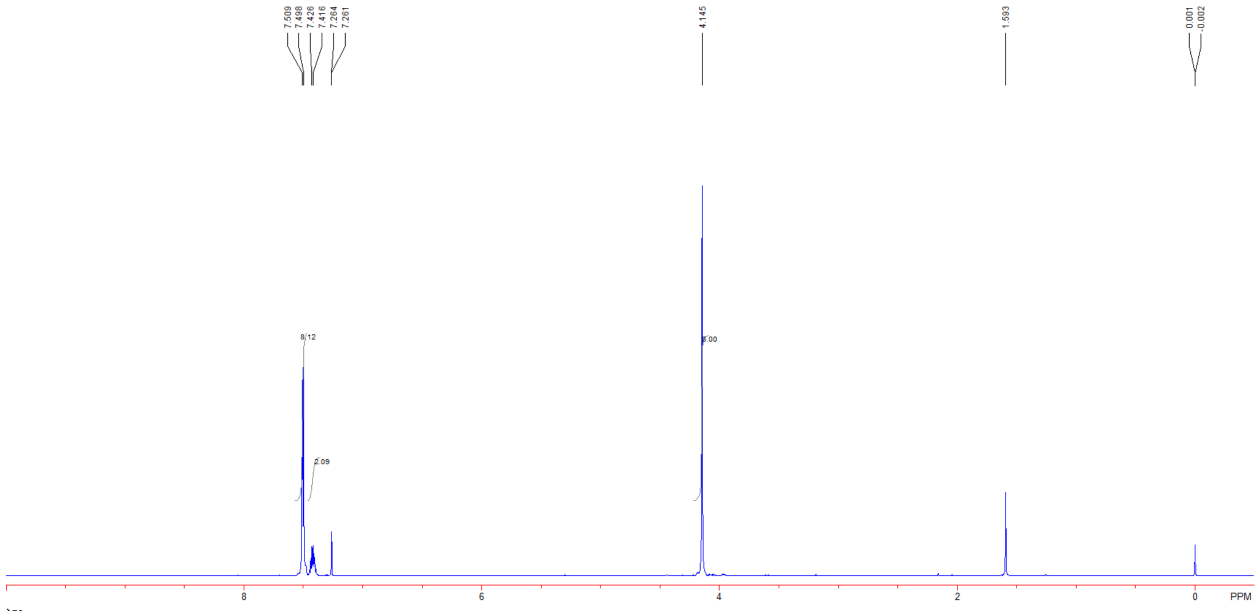

$^{13}C\{^{1}H\}$ NMR Spectrum of Compound 7 in $CDCl_3$ (100 MHz)

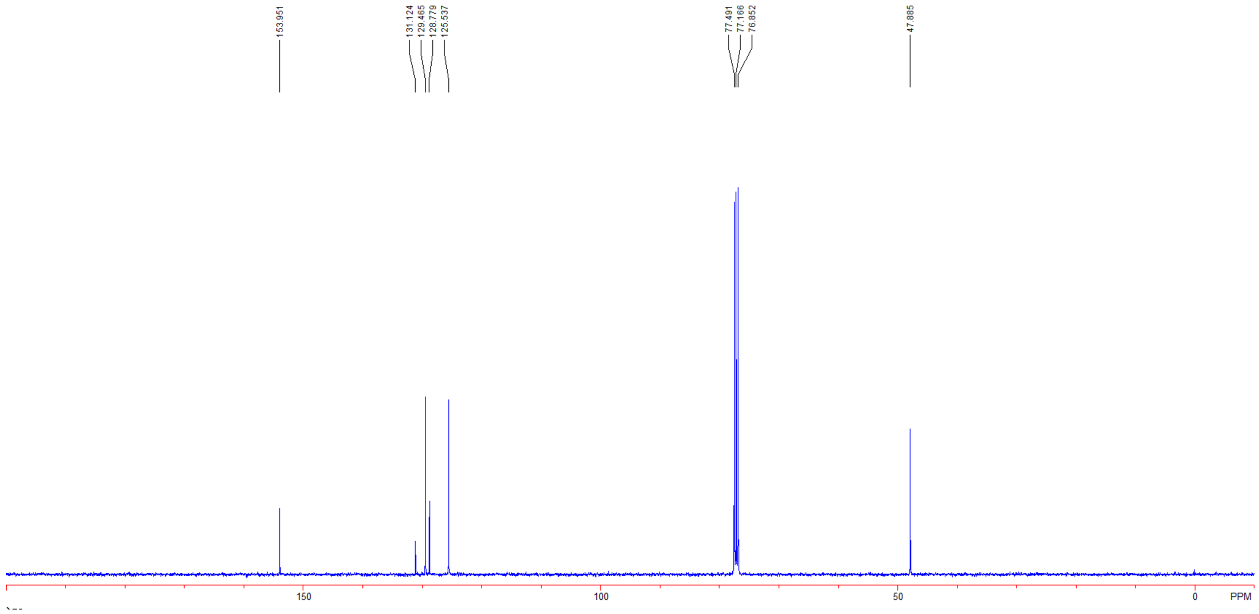

^{1}H NMR Spectrum of Compound 11 in $CDCl_3$ (400 MHz)

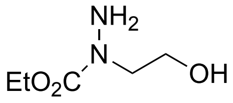

11

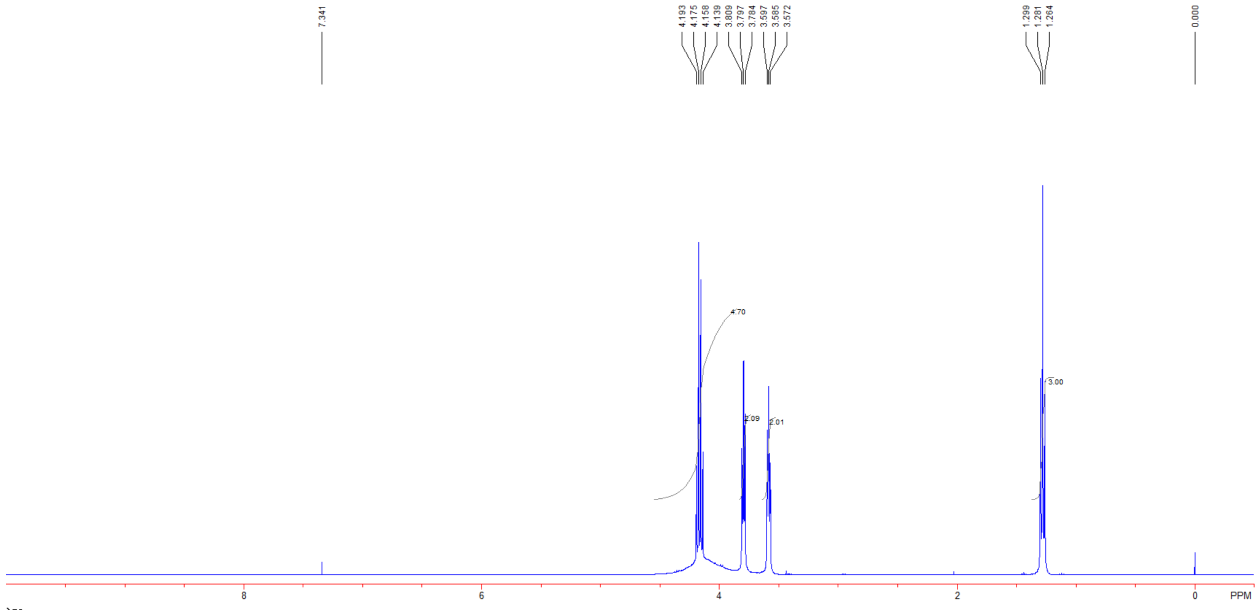

^{13}C{^{1}H} NMR Spectrum of Compound 11 in $CDCl_3$ (100 MHz)

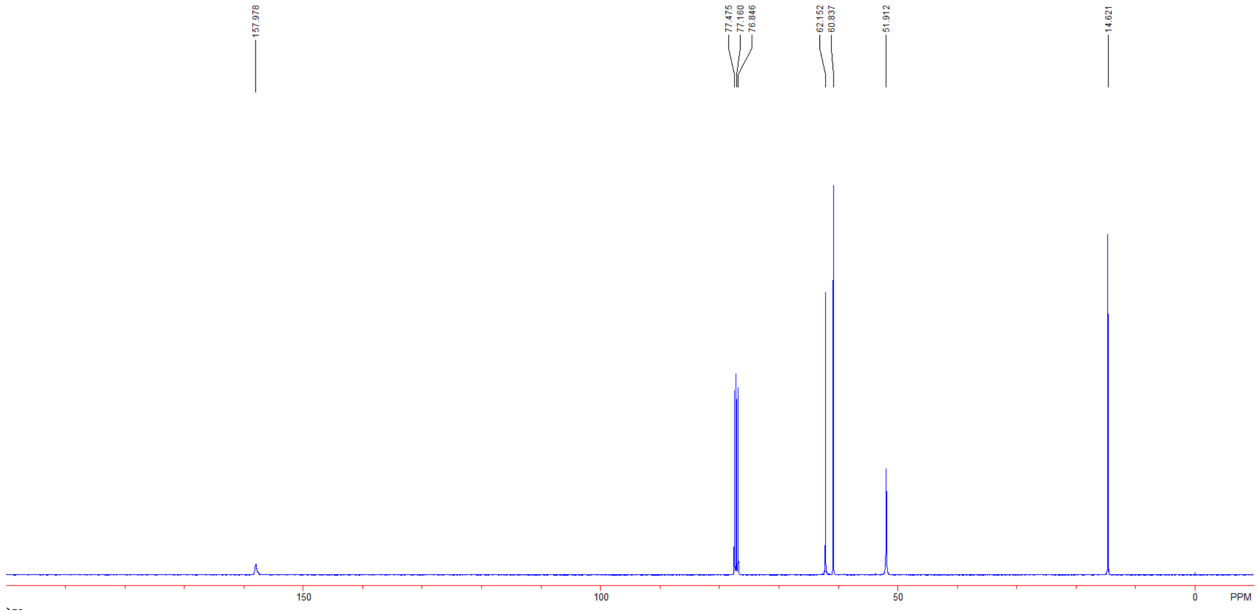

^{1}H NMR Spectrum of Compound 12 in DMSO-D$_6$ (400 MHz)

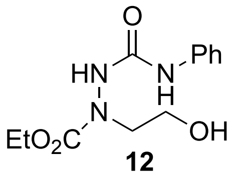

12

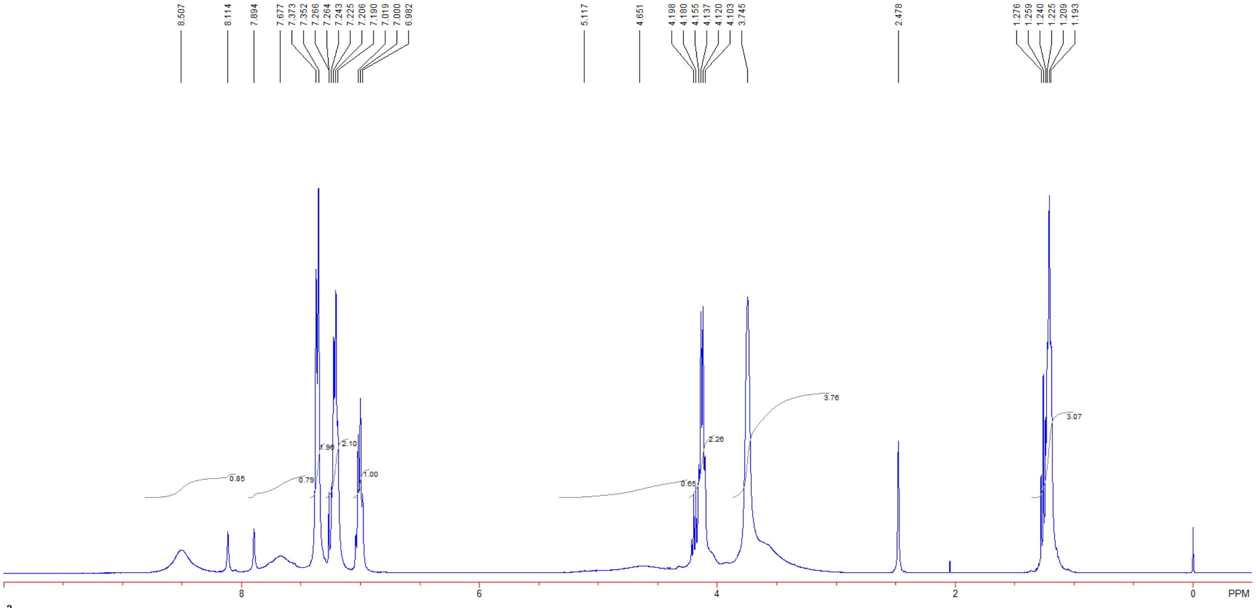

^{13}C{^{1}H} NMR Spectrum of Compound 12 in CDCl$_3$ (100 MHz)

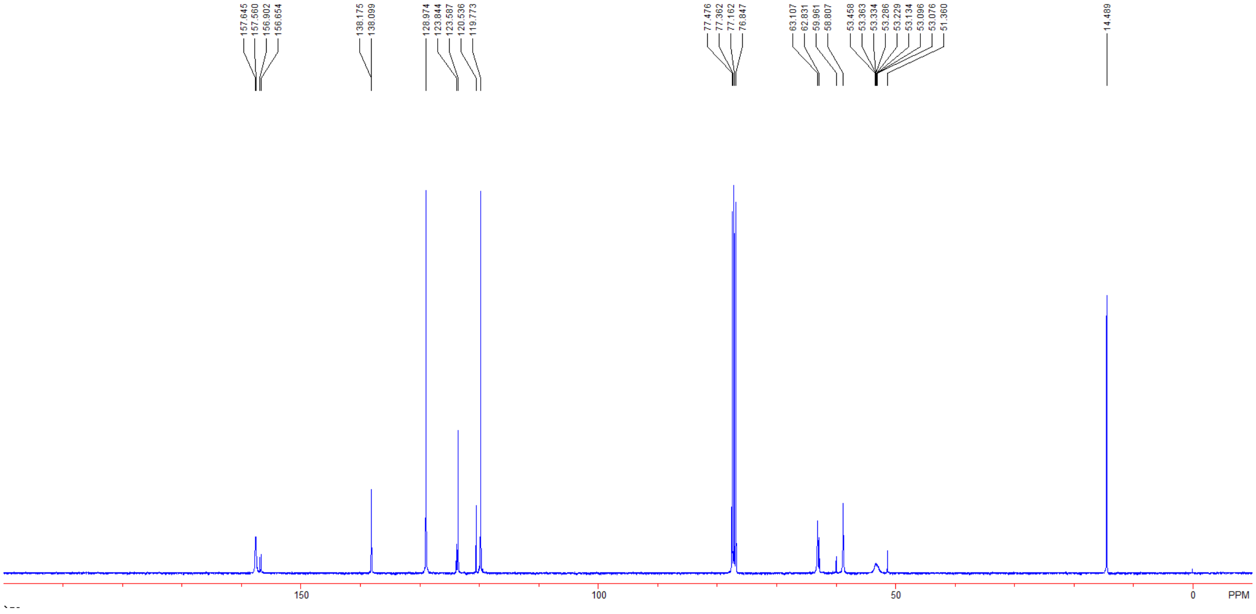

^{1}H NMR Spectrum of Compound 13 in DMSO-D$_6$ (400 MHz)

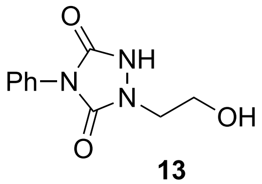

13

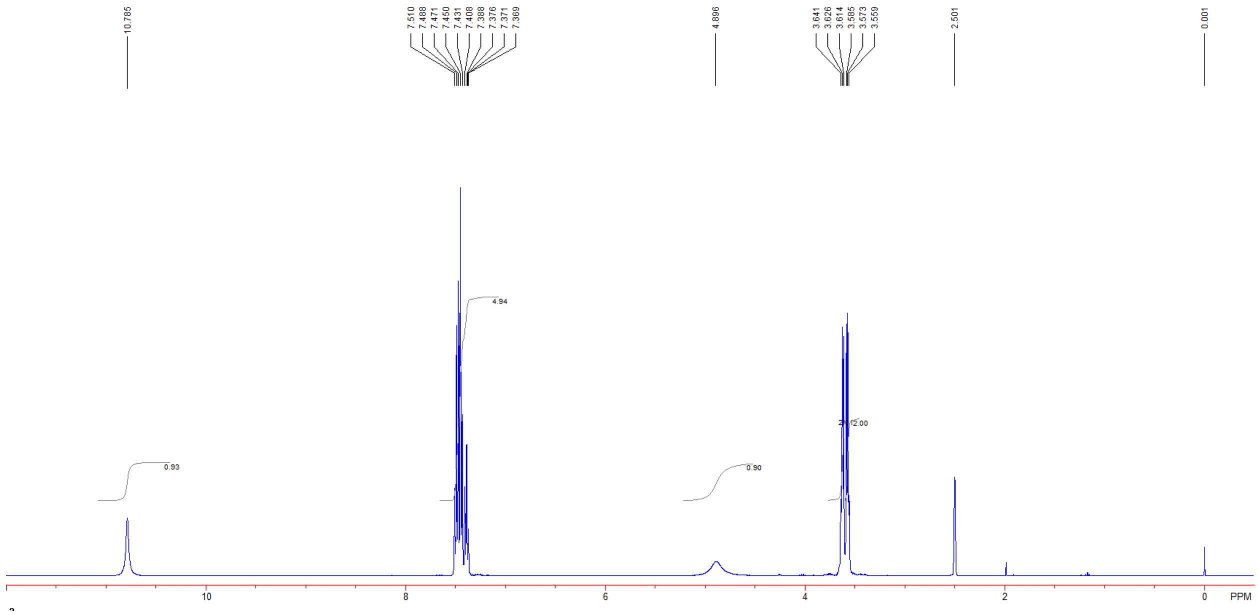

^{13}C{^{1}H} NMR Spectrum of Compound 13 in DMSO-D$_6$ (100 MHz)

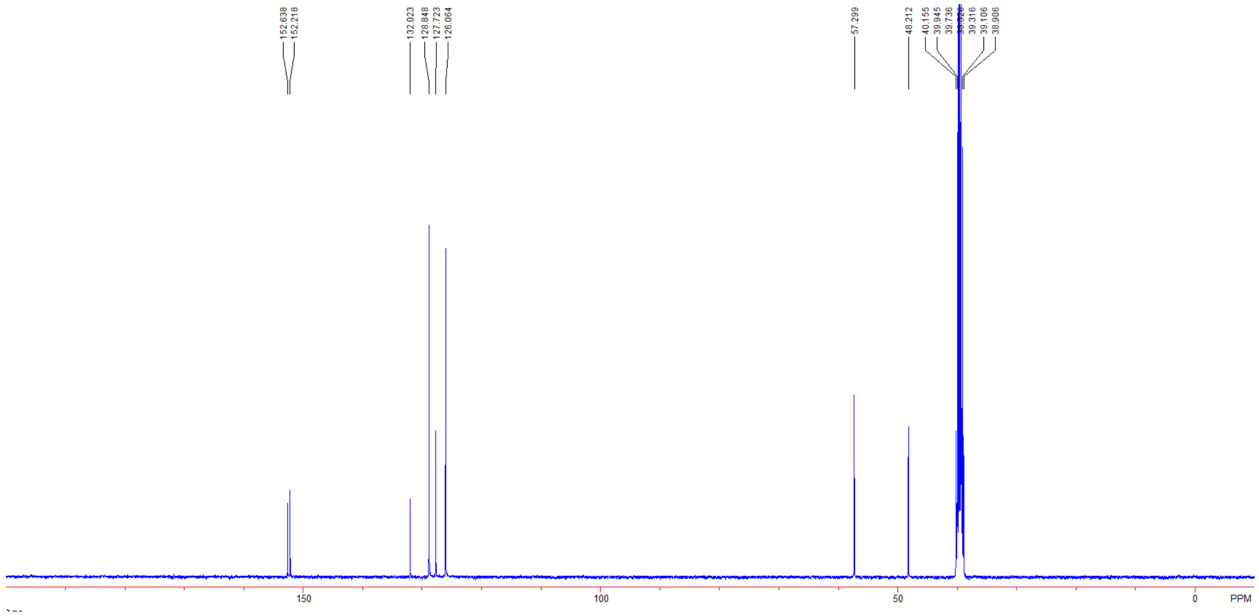

^{1}H NMR Spectrum of Compound 14 in DMSO-D$_6$ (400 MHz)

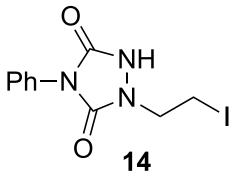

14

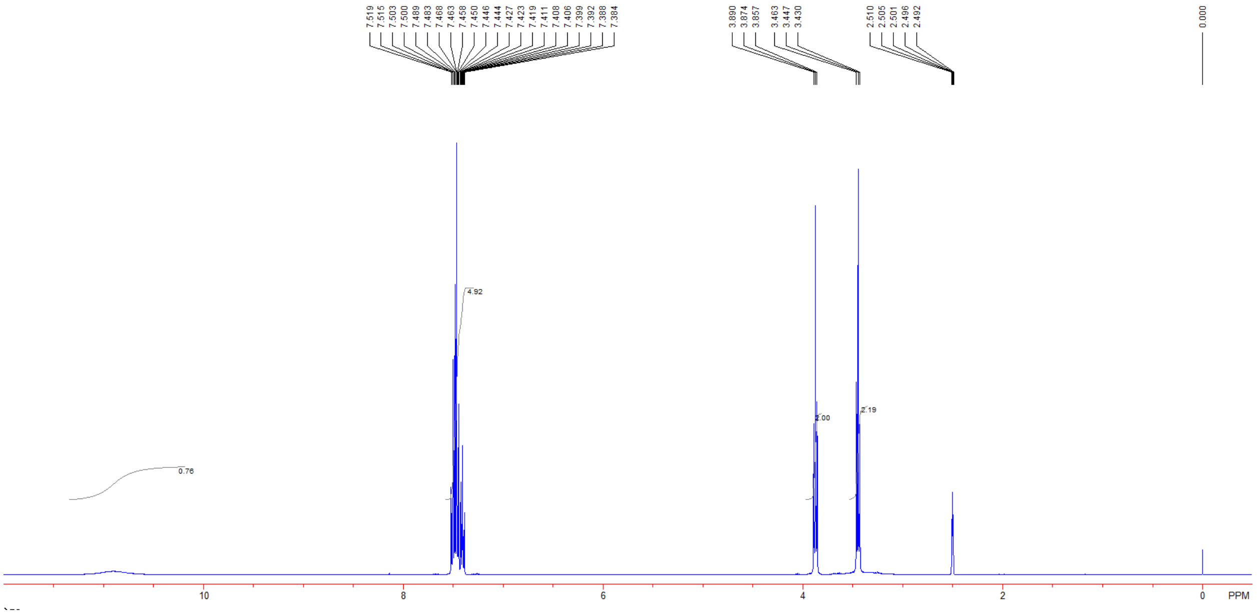

^{13}C{^{1}H} NMR Spectrum of Compound 14 in DMSO-D$_6$ (100 MHz)

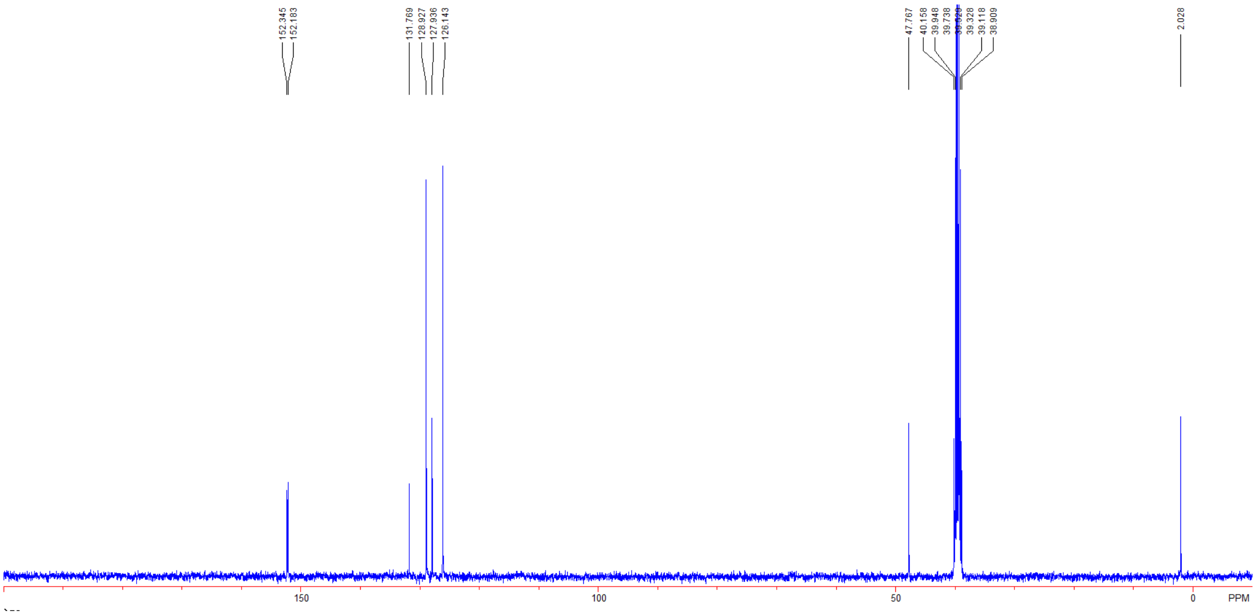

^{1}H NMR Spectrum of Compound 3 in $CDCl_3$ (400 MHz)

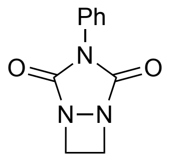

3

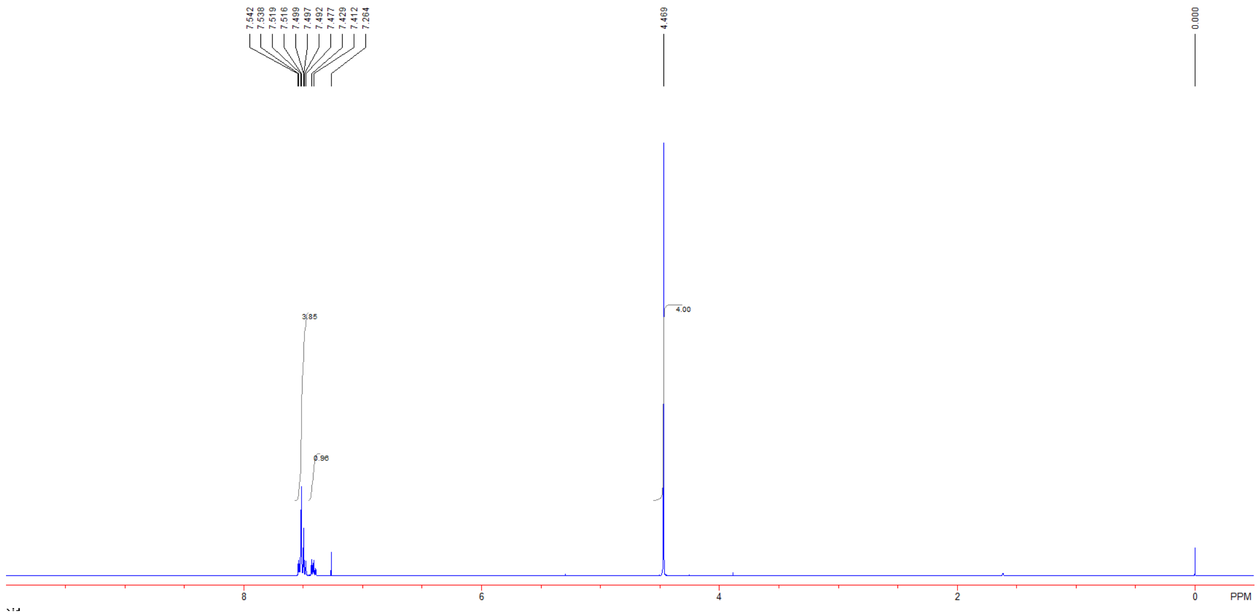

$^{13}C\{^{1}H\}$ NMR Spectrum of Compound 3 in $CDCl_3$ (100 MHz)

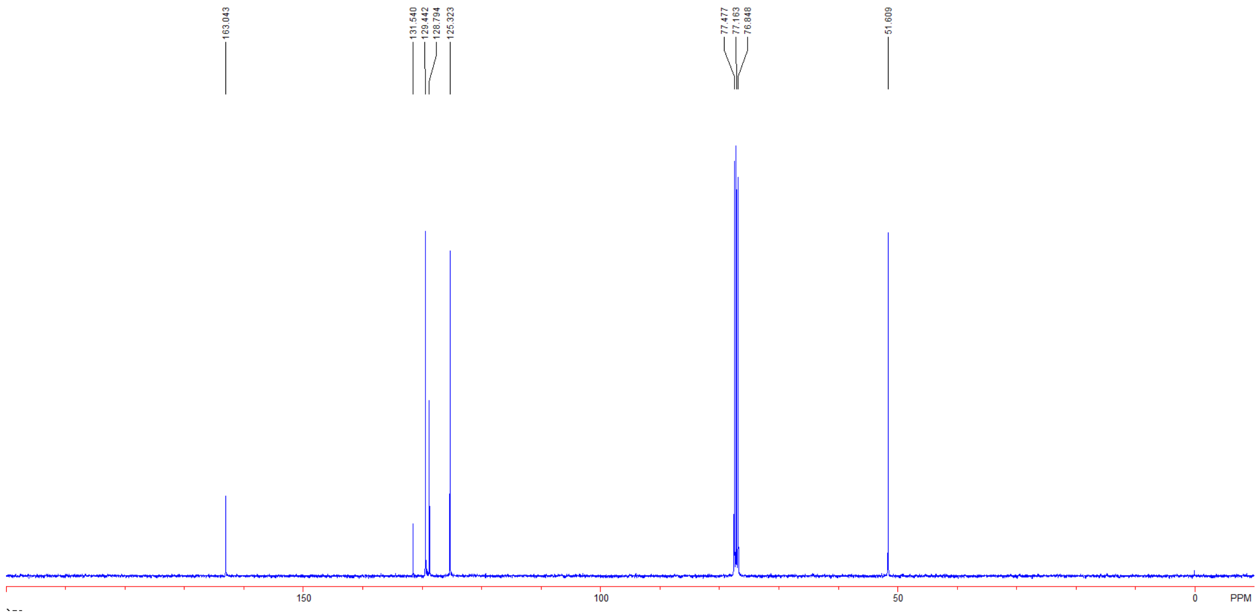

Supplement: Supplementary file 1 [file molecules-29-04068-s001.zip › molecules-3156785-supplementary.pdf]
